# Effects of galloyl group on the astringency perception of epigallocatechin gallate and epigallocatechin

**DOI:** 10.1016/j.crfs.2025.101120

**Published:** 2025-06-18

**Authors:** Zhenyu Zhou, Qian Huang, Wangyang Shen, Weiping Jin, Guoyan Yang, Wenjing Huang, Cheng Guo

**Affiliations:** a College of Food Science and Engineering, Wuhan Polytechnic University, Wuhan, 430023, China; b Key Laboratory for Deep Processing of Major Grain and Oil (Wuhan Polytechnic University), Ministry of Education, Wuhan, 430023, China; c Hubei Key Laboratory for Processing and Transformation of Agricultural Products, Wuhan Polytechnic University, Wuhan, 430023, China

**Keywords:** Epigallocatechin gallate, Epigallocatechin, Salivary protein, Molecular interaction, Salivary film, Astringency receptor

## Abstract

The astringency in green tea primarily originates from gallate-type and non-gallate-type catechins. Epigallocatechin gallate (EGCG) is a gallate-type catechin, which has a strong astringency and is the most abundant catechin in tea. Unlike EGCG, epigallocatechin (EGC) has one fewer galloyl group on the C3 oxygen atom and exhibits a weaker astringency. Taking EGCG and EGC as representative catechins, this study investigated the effects of the galloyl group on their astringency. The interactions of EGCG and EGC with salivary proteins were qualitatively and quantitatively analyzed using fluorescence spectroscopy, isothermal titration calorimetry, and molecular docking. The surface roughness and viscoelasticity of the salivary film were then studied to relate the molecular interactions and perceived astringency. Additionally, the bindings of EGCG and EGC to astringency receptor proteins were also simulated. The results revealed that the galloyl group enabled the EGCG-promoted aggregation of salivary proteins, resulting in a stronger astringency through both tactile and gustatory pathways. In contrast, EGC exhibited a weaker astringency only through the gustatory pathway.

## Highlights

• Galloyl group enabled the EGCG-promoted aggregation of salivary proteins.
• The binding abilities of EGCG and EGC to salivary proteins was evaluated.
• Rheology was used to relate molecular interactions and perceived astringency.
• The bindings of EGCG and EGC to astringency receptors were simulated.
• The differences in astringency perception mechanisms of EGCG and EGC was proposed.

## Graphical abstract

**Figure:**
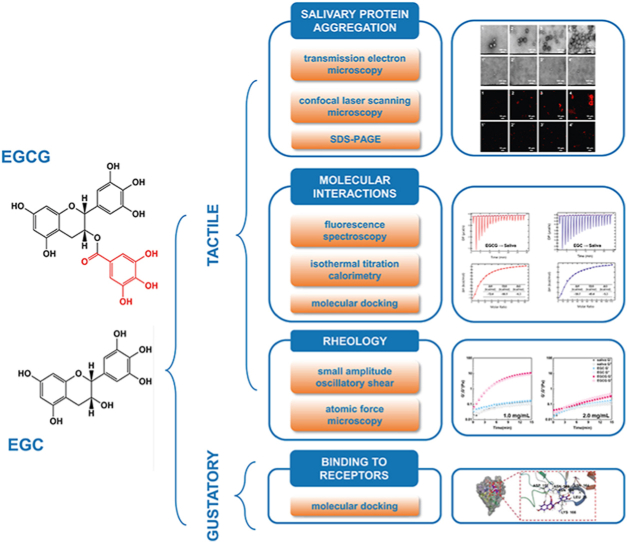

## 1 Introduction

**Fig. 1:**
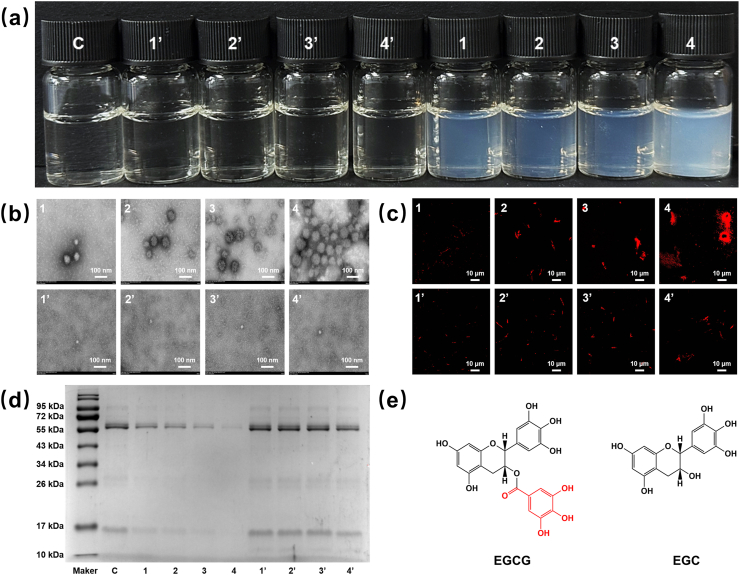
(a) Saliva after adding EGCG or EGC. C: saliva, 1: EGCG (0.25 mg/mL), 2: EGCG (0.5 mg/mL), 3: EGCG (1.0 mg/mL), 4: EGCG (2.0 mg/mL), 1′: EGC (0.25 mg/mL), 2′: EGC (0.5 mg/mL), 3′: EGC (1.0 mg/mL), 4′: EGC (2.0 mg/mL). (b) Transmission electron microscopy images of the mixed samples. (c) Confocal laser scanning microscopy images of the mixed samples. (d) Electrophoretic pattern of proteins in the supernatant of the mixed samples. (e) The molecular structure of EGCG and EGC.

Tea is one of the most widely consumed beverages globally, with astringency being a key characteristic that defines its flavor. The astringency of green tea mainly comes from catechins (Xu et al., 2018). Catechins are a class of polyphenols found in the tea leaves and buds, which can be classified into gallate-type and non-gallate-type by the presence of a galloyl group on the C3 oxygen atom (Hayashi et al., 2004). Epigallocatechin gallate (EGCG) is a gallate-type catechin, which has a strong astringency and is the most abundant catechin in tea (Khalatbary and Khademi, 2020; Ye et al., 2021). Unlike EGCG, epigallocatechin (EGC) has one fewer galloyl group in its structure and exhibits a weaker astringency (Fig. 1e) (Ferrer-Gallego et al., 2014; Huang and Xu, 2021). Although gallate-type catechins have higher physiological activities than non-gallate-type catechins, it is unclear how differences in their molecular structure affect their astringency.

At present, there are two generally accepted pathways to perceive the astringency of polyphenols. In the tactile stimulus pathway, polyphenols and salivary proteins combine through non-covalent bonds, changing the structure and physical properties of the salivary film. The lubricating properties of the saliva are partially lost, resulting in the dryness, stickiness, and roughness of the salivary film (Wang et al., 2021). With the increase of polyphenol concentration, large particles of insoluble polyphenol–protein aggregates precipitate, which exceeds the bearing limit of salivary film, leading to film rupture and the significant loss of lubrication properties. The large aggregates are directly exposed to the oral surface, resulting in increased friction and astringency (Gibbins and Carpenter, 2013; Huang and Xu, 2021).

In the gustatory pathway, an astringent sensation results from the direct interaction of polyphenols with oral receptors. Catechins such as EGCG and EGC can activate the hTAS2R39 human taste receptor, while trigeminal neurons can directly respond to astringency without friction stimulation (Narukawa et al., 2011; Schöbel et al., 2014). Other studies have identified the 67 kDa laminin receptor (67LR) protein as a potential oral cell receptor involved in the sensation of phenolic compounds. Tea polyphenols can activate transient receptor protein channels to promote the Ca^2+^ response, thereby contributing to the perceived astringency (Kurogi et al., 2012).

Thus, we proposed that the galloyl group increases the steric hindrance and complexity of catechin molecules, altering their interaction with salivary proteins and binding to astringency receptors, leading to differences in the perceived astringency of gallate-type and non-gallate-type catechins.

In this study, EGCG and EGC were selected as representative gallate-type and non-gallate-type catechins, respectively. First, the EGCG- and EGC-induced aggregation of salivary proteins was compared using transmission electron microscopy, confocal laser scanning microscopy (CLSM), and sodium dodecyl sulfate–polyacrylamide gel electrophoresis (SDS-PAGE). Second, the molecular mechanism underlying the differences in salivary protein aggregation was explored by fluorescence spectroscopy and isothermal titration calorimetry (ITC). Third, the effects of EGCG and EGC on the surface structure and rheology of the salivary film were further characterized by atomic force microscopy (AFM) and small amplitude oscillatory shear (SAOS) rheology.

Additionally, differences in the binding of EGCG and EGC with astringency receptors were compared using in-silico technique. Nowadays, in-silico technique has been widely applied in life sciences research and has achieved significant progress. It simulates the dynamic behavior of biomolecules, intracellular signaling processes, and the complex interactions within biological systems by constructing mathematical models and utilizing high-performance computing (Alsahag, 2025; Kamaraj et al., 2013; Kumar et al., 2014). Molecular docking is considered a highly reliable computational tool that can determine the positioning of ligand molecules at the active sites of target proteins and elucidate the precise mechanisms of selective binding of substrates or inhibitors (Sharma et al., 2023). Here, the molecular docking was employed to investigate the binding sites and binding affinities of the two types of catechins with astringency receptor proteins.

Based on these results, we analyzed the effects of the galloyl group on catechin–salivary protein interactions and catechin–astringency receptor binding. By identifying connections between these micromolecular interactions and macroscopic changes in the salivary film structure and rheology, we can elucidate the molecular mechanism causing the differences in astringency perception between gallate-type and non-gallate-type catechins.

## 2 Materials and methods

### 2.1 Materials

EGCG (≥98 %) and EGC (≥98 %) were purchased from Aladdin Biochemical Technology Co., Ltd. (Shanghai, China). All other analytical-grade reagents were purchased from Sinopharm Chemical Reagents Co., Ltd. (Shanghai, China). Milli-Q purified water was used in all experiments.

### 2.2 Saliva collection

Four volunteers (three males and one female) aged 22–24 years, who had no history of smoking, drinking, or oral diseases, were selected for the study (Agorastos et al., 2024). Fresh, unstimulated saliva was collected from 10:00 to 11:00 a.m., with the volunteers instructed to abstain from any food or water consumption at least 2 h prior to the saliva collection process (Vijay et al., 2015). The collected saliva was mixed, placed in a centrifuge tube, and immediately centrifuged at 10000 *g* for 10 min to remove any insoluble substances (Agorastos et al., 2024; Zhou et al., 2024).

### 2.3 Sample preparation

EGCG and EGC powders were dissolved in PBS solution (100 mM, pH 6.0) to prepare the EGCG and EGC solutions. The EGCG and EGC solutions were mixed with the non-precipitable saliva fraction at a 1:1 vol ratio to prepare the EGCG/saliva and EGC/saliva mixed samples. Considering the EGCG and EGC astringency perception thresholds of 0.086 and 0.16 mg/mL (Huang and Xu, 2021), respectively, and the EGCG content in green tea extract being generally not higher than 2.0 mg/mL (Zuo et al., 2002), the concentrations of EGCG and EGC in the mixed samples were set to 0.25, 0.5, 1.0, and 2.0 mg/mL.

### 2.4 Transmission electron microscopy

A drop (10 μL) of the mixed sample was applied to a carbon-coated grid that had been glow discharged for 1 min in air, and the grids were immediately negatively stained using 2 % phosphotungstic acid for 60 s. The grids were examined on an H-7800 transmission electron microscope (HITACHI Inc., Japan) operated at 80–120 kV.

### 2.5 Confocal laser scanning microscopy

The samples were held at 25 °C for 10 min. Then, 90 μL of the sample was pipetted into a PCR tube, and 10 μL of 0.2 % rhodamine B was added. The mixed solution was stained at room temperature in the dark for 20 min (Reddy et al., 2022). Then, 10 μL of the stained sample was placed on a slide and imaged using a confocal laser scanning microscope (FV300, Olympus Inc., Japan).

### 2.6 Sodium dodecyl sulfate–polyacrylamide gel electrophoresis

Briefly, 10 mL of the mixed sample was maintained at 25 °C for 15 min and centrifuged at 10,000 *g* for 10 min to isolate the supernatant. Then, 40 μL of the supernatant was mixed with 10 μL of the loading buffer (consisting of 50 mM Tris-HCl pH 6.8, 100 mM β-mercaptoethanol, 1 % SDS, 0.0025 % bromophenol blue, and 10 % glycerol) and incubated at 95 °C for 5 min. The resolving and stacking gels had acrylamide concentrations of 12 % and 5 %, respectively. The molecular weights were estimated by comparison with the migration rates of the BeyoColor™ prestained color protein marker (10–180 kDa) (Zhou et al., 2024).

### 2.7 Mass spectrometry

The liquid chromatography–tandem mass spectrometry (LC-MS/MS) identification and data analysis were performed by SpecAlly Life Technology Co., Ltd. (China). Briefly, the sample was mashed with a glass rod and mixed with ddH_2_O, decolorizing solution, and acetonitrile (ACN) for decolorization. The sample was vortexed for 5 min and centrifuged, discarding the supernatant. An appropriate amount of trypsin was added according to the sample volume for overnight digestion at 37 °C. The next day, the peptide extraction solution (60 % ACN/5 % formic acid) was added, and the mixture was sonicated for 10 min, centrifuged to remove the supernatant, and vacuum dried. The sample was desalted using a C18 desalting column, vacuum dried, and stored at −20 °C for later use. LC-MS/MS data acquisition was performed on a Q Exactive HF mass spectrometer coupled with an UltiMate 3000 RSLCnano system (Thermo Fisher Scientific Inc., USA). The peptides were loaded through an auto-sampler and separated in a C18 analytical column (75 μm × 25 cm, C18, 1.9 μm, 120 Å). Mobile phases A (0.1 % formic acid) and B (80 % ACN, 0.1 % formic acid) were used to establish the separation gradient at a constant flow rate of 300 nL/min. For the data-dependent acquisition analysis, each scan cycle consisted of one full-scan mass spectrum (R = 60 K, AGC = 3e6, max IT = 25 ms, scan range = 350–1500 m/z) followed by 20 MS/MS events (R = 15 K, AGC = 1e5, max IT = 50 ms). The higher-energy collision dissociation was set to 27, the isolation window for precursor selection was set to 1.4 Da, and the former target ion exclusion was set for 24 s. The MS raw data were analyzed with MaxQuant (V1.6.6) using the Andromeda database search algorithm. Spectra files were searched against the SwissProt Human protein database, and the search results were filtered with a 1 % false discovery rate at the protein and peptide levels (Zhou et al., 2024).

### 2.8 Fluorescence spectroscopy

A fluorescence spectrophotometer (F-4600, HITACHI, Japan) was used to measure the binding of EGCG and EGC with salivary proteins. The EGCG and EGC powders were dissolved in PBS solution (100 mM, pH 6.0), and the concentrations of EGCG and EGC were fixed at 3 mM. Varying volumes of EGCG or EGC solutions (10, 20, 30, 40, 50, and 60 μL) were individually added to 90 μL of saliva. The total volume was fixed to 3 mL with PBS solution, and the fluorescence intensity was measured immediately at 25 °C, the mean of three replicates were calculated. The emission spectra were recorded at an excitation wavelength of 280 nm, with both the excitation and emission slit widths set to 5 nm (Zhan et al., 2018).

### 2.9 Molecular docking

The crystal structures of α-amylase (PDB code 1JXJ), lipocalin 1 (PDB code 1XKI), and the 67LR protein (PDB code 3BCH) were obtained from the Protein Data Bank (https://www.rcsb.org/). The crystal structure of the hTAS2R39 human taste receptor protein was generated by AlphaFold3, while the crystal structure of cystatin S (AF-P01036-F1-model_v4) was taken from the Universal Protein Resource (https://www.uniprot.org/). Protein preprocessing was performed on the obtained protein crystals using the Protein Preparation Wizard module in Schrödinger. The two-dimensional (2D) SDF structure files of EGCG and EGC obtained from the PubChem database (https://pubchem.ncbi.nlm.nih.gov/) were optimized under the OPLS4 force field using the LigPrep module in Schrödinger to generate their respective 3D chiral conformations. The SiteMap module in Schrödinger was then used to predict the optimal binding sites between the ligand and protein. The Receptor Grid Generation module in Schrödinger was used to set the most appropriate enclosing box (30 × 30 × 30 Å) to perfectly wrap the predicted binding sites, and the protein active sites were obtained on this basis. Next, molecular docking (i.e., XP docking with the highest precision) of the ligand compounds with the protein active sites was performed. The lower XP GScore indicated the lower binding free energy and higher binding stability of the compound to the protein. The interactions between the ligand compounds and protein active sites were analyzed through molecular mechanics with generalized Born surface area (MM-GBSA) calculations. The MM-GBSA calculations were performed on minimized complexes using the Prime MM-GBSA module in Schrödinger. MM-GBSA dG Bind can approximately represent the ligand–protein binding free energy (Dong and Li, 2024; Teng et al., 2023).

### 2.10 Isothermal titration calorimetry

ITC determination was performed at 25 °C using a MicroCal PEAQ-ITC microcalorimeter (MicroCal Inc., USA). Briefly, 40 μL of EGCG (2.2 mM) solution or EGC (3.2 mM) solution was injected sequentially into a 200 μL titration cell containing saliva. The EGCG and EGC solutions were both prepare using PBS solution (100 mM, pH 6.0). The titration was performed with 19 successive 2 μL injections, with the first 0.5 μL injection not counted in the result. Each addition lasted 60 s with an interval of 90 s between successive injections. The stirring speed was set at 750 rpm. The calibration experiment was conducted with an EGCG (2.2 mM) or EGC (3.2 mM) titration of PBS solution (100 mM, pH 6.0), and the heat released during the calibration was subtracted from the raw data (Zhou et al., 2024).

### 2.11 Small amplitude oscillatory shear rheology

The rheological properties of the samples were determined with an AR-500 dynamic rheometer (TA Instruments Inc., USA) at 25 °C using a 40 mm plate geometry. The freshly prepared samples were transferred to a rheometer and set for 3 min. The SAOS tests were conducted at 2 % strain with an oscillatory shear frequency of 0.1 Hz (Zhou et al., 2024).

### 2.12 Atomic force microscopy

The samples after ultrasonic dispersion were dropped onto a silicon wafer and dried. The surface morphologies of the samples were measured in the tapping mode using an atomic force microscope (Dimension® Icon™, Bruker Inc., USA) at a scan frequency of 1 Hz and a scan area of 10 μm × 10 μm. The number of images per sample was 7, and the Nanoscope Analysis software V3.00 (Bruker Inc., USA) was used to quantify roughness metrics.

### 2.13 Statistical analysis

Statistical analysis was performed using SPSS statistical software V22.0 (IBM, USA), and the mean and standard deviation of three replicates were calculated. Statistically significant differences were measured using the one-way analysis of variance followed by the Tukey multiple comparison test, with *p* < 0.05 representing statistically significant differences.

## 3 Results and discussion

### 3.1 Differences between EGCG and EGC in promoting salivary protein aggregation

The catechins promoted salivary protein aggregation, which was the primary cause of their perceived astringency (Shih et al., 2017). EGCG and EGC showed significant differences in promoting salivary protein aggregation. As EGCG was added, an increasing amount of white turbidity formed in the saliva (Fig. 1a). In contrast, EGC did not cause the saliva to become turbid within the range of the added amount in this experiment. Transmission electron microscopy indicated that spherical aggregates formed in the saliva after the addition of EGCG, whereas no aggregation was observed after the addition of EGC (Fig. 1b). Salivary proteins in the samples were then stained with rhodamine B and observed by CLSM (Fig. 1c). Spherical aggregates formed from the aggregation of salivary proteins, the extent of which was proportional to the concentration of EGCG. Previous studies have shown that at low polyphenol concentrations, salivary proteins and polyphenols will aggregate to form small, soluble polydisperse particles. With increasing polyphenol concentration, some polyphenols can act as bridging agents for protein molecules, resulting in protein aggregation and precipitation (Berkel Kasikci et al., 2024; Siebert et al., 1996). Furthermore, the supernatant of the mixed samples after centrifugation was determined by SDS-PAGE (Fig. 1d). As the concentration of EGCG increased gradually from 0.25 to 2.0 mg/mL, the protein bands (55, 26, and 17 kDa) became progressively lighter, while EGC had no significant effect on the protein bands. Compared with EGC, EGCG can promote the aggregation of salivary proteins, facilitating their removal by centrifugation. The interactions between EGCG and salivary proteins induce conformational changes in the proteins, exposing additional binding sites. This allows the proteins to bind with more EGCG molecules, leading to aggregation (Zhou et al., 2015).

### 3.2 Interactions of EGCG and EGC with salivary proteins

**Fig. 2:**
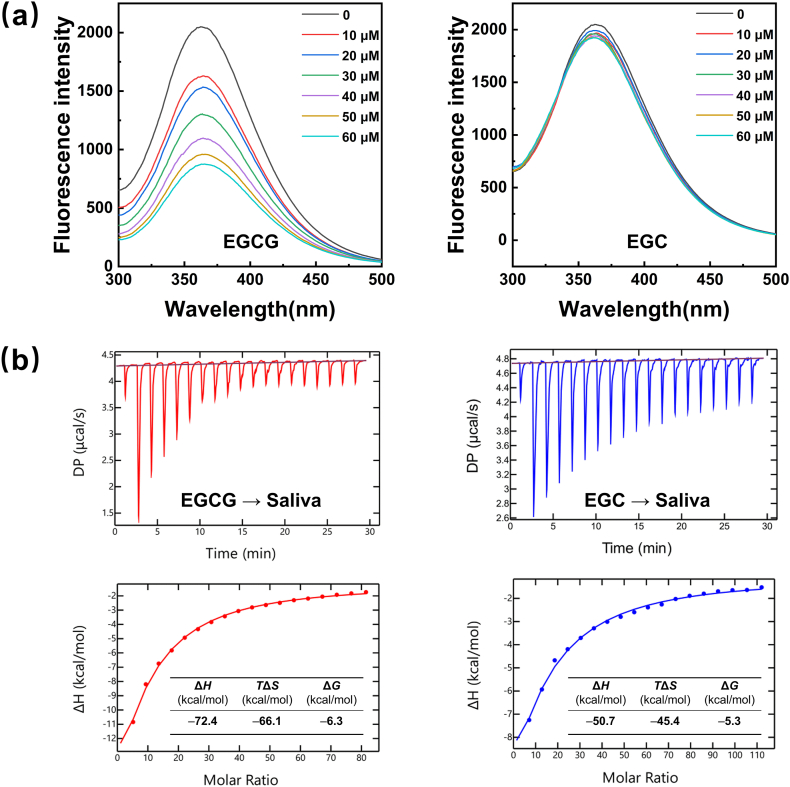
(a) Effect of EGCG and EGC on the fluorescence emission spectra of salivary proteins. (b) Thermograms (upper panels) and binding isotherms (lower panels) corresponding to the titration of saliva with EGCG (2.2 mM) and EGC (3.2 mM) solution.

The interactions of EGCG and EGC with salivary proteins are the molecular basis for the differences in salivary protein aggregation. The binding of catechins to salivary proteins led to protein fluorescence quenching, reflecting the changes in the protein structure and microenvironment (Soares et al., 2007). Tyrosine and tryptophan residues in proteins exhibit fluorescent properties, producing fluorescence emissions at an excitation wavelength of 280 nm (Yang et al., 2008; Zhan et al., 2018). Fig. 2a shows the fluorescence emission spectra of salivary proteins excited at 280 nm for different concentrations of EGCG and EGC. The intensity of the fluorescence emission of salivary proteins at 360 nm regularly decreased with increasing EGCG concentration. However, the fluorescence emission intensity did not change significantly when EGC was added. EGCG could more effectively approach and bind to the fluorescent chromophores of salivary proteins, resulting in a more obvious fluorescence quenching phenomenon. In contrast, the binding ability of EGC to salivary proteins was weak and had little impact on the fluorescent chromophores.

The differences in the interactions of EGCG and EGC with salivary proteins were quantitatively characterized by ITC. In Fig. 2b, the smooth curves were well fitted with the experimental data points, suggested the “one set of sites” model was suitable for the experiment. The downward titration curve indicated that an exothermic reaction occurred when EGCG or EGC was titrated into saliva. The thermodynamic parameters (Δ*G*, Δ*H*, and *T*Δ*S*) of these interactions were negative, suggesting the spontaneity of these primarily noncovalent interactions (Rudge et al., 2021; Zhan et al., 2018). Moreover, the absolute values of Δ*H* and TΔ*S* for the titration of saliva with EGCG were significantly higher than those for the titration of saliva with EGC, indicating that the binding ability of EGCG to salivary proteins was stronger than that of EGC.

**Fig. 3:**
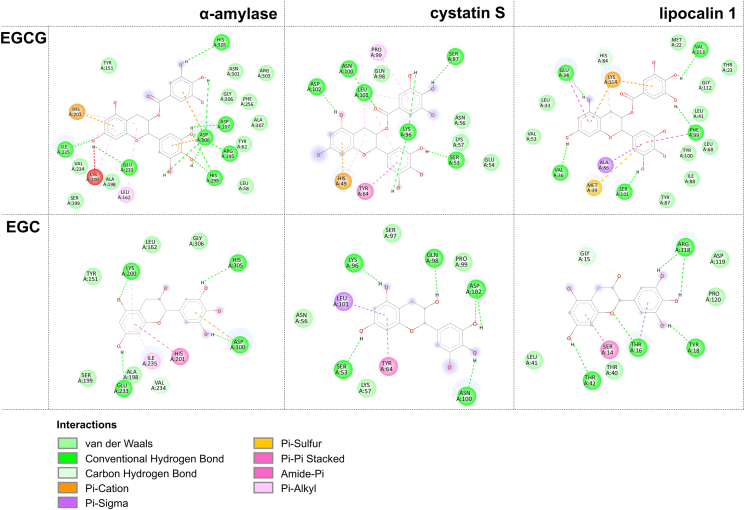
The 2D patterns of the interfacial interactions between EGCG/EGC and α-amylase/cystatin S/lipocalin 1.

Furthermore, the binding sites and binding modes of EGCG or EGC with salivary proteins were investigated via molecular docking. LC-MS/MS identification of the SDS-PAGE protein bands revealed that the salivary proteins α-amylase (55 kDa), lipocalin 1 (26 kDa), and cystatin S (17 kDa) were readily aggregated and precipitated by EGCG, which was consistent with the results of previous studies (Hara et al., 2012; Zhou et al., 2024). The binding sites and interaction forces of EGCG and EGC with the three proteins are shown in Fig. 3 and Table S1–3. EGCG interacted with 21, 14, and 19 amino acid residues of α-amylase, cystatin S, and lipocalin 1, respectively, while EGC had much fewer binding sites and interacted with only 12, 11, and 10 amino acid residues of these proteins.

In summary, the interaction between EGCG and the salivary proteins was significantly stronger than that of EGC due to differences in molecular structure. EGCG contains more phenolic hydroxyl and galloyl groups, which can form additional hydrogen bonds and hydrophobic interactions with the carbonyl and imidazole residues of salivary proteins. This enables EGCG to bind more extensively with salivary proteins, inducing cross-linking between protein molecules (Yamada et al., 2007). The galloyl group increased the molecular complexity and hydrophobicity, allowing EGCG to interact with the salivary proteins not only through hydrogen bonding via phenolic hydroxyl groups but also through hydrophobic interactions, resulting in stronger protein bonding. The hydrophobic groups of EGCG interacted with the hydrophobic regions of the salivary proteins to form a relatively stable hydrophobic core. This caused the surrounding protein molecules to continuously gather around it, forming aggregates and precipitating (Poncet-Legrand et al., 2006). The molecular structure of EGC is relatively simple and lacks a galloyl group. The binding of EGC to salivary proteins is primarily achieved through the formation of hydrogen bonds between the phenolic hydroxyl groups and polar groups on the protein surface, which results in fewer binding sites with salivary proteins, a relatively singular mode of interaction, and a weaker binding affinity. Thus, although EGC can bind to salivary proteins, it cannot sufficiently induce cross-linking and aggregation between protein molecules.

### 3.3 Effects of EGCG and EGC on the salivary film

Salivary proteins are extremely surface active and adsorb to the air–liquid interface to form a high-elasticity “solid-like” surface film (Rossetti et al., 2008). The oral surface is covered by a salivary film, which plays an important role in oral lubrication. The interactions between salivary proteins and catechins affect the structure and physical properties of the salivary film, leading to increased roughness. The intensity of astringency is strongly positively correlated with the roughness of the salivary film (Lei et al., 2022). Therefore, the ability of catechins to alter the physical properties of the salivary film is a key factor contributing to the astringency they produce.

**Fig. 4:**
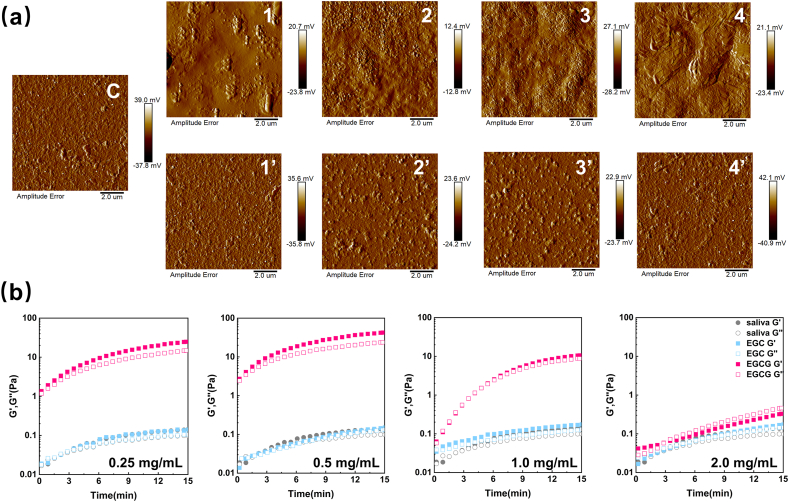
Atomic force microscopy images of the mixed samples. C: saliva, 1: EGCG (0.25 mg/mL), 2: EGCG (0.5 mg/mL), 3: EGCG (1.0 mg/mL), 4: EGCG (2.0 mg/mL), 1′: EGC (0.25 mg/mL), 2′: EGC (0.5 mg/mL), 3′: EGC (1.0 mg/mL), 4′: EGC (2.0 mg/mL). (b) Dynamic changes of the elasticity modulus (G′) and the viscosity modulus (G″) with time.

The changes in the surface morphology of salivary film after the addition of catechins were observed by AFM. AFM imaging has become an important tool for studying the structure and dynamics of membrane proteins in their physiological environment at high resolution, generating high-resolution images of membrane proteins related to the oral sensation of food (Koehler et al., 2024). Fig. 4a shows the surface morphology of the salivary film in the presence of different concentrations of EGCG and EGC. The salivary film was composed of a dense inner layer with small protrusions uniformly dispersed in the outer layer (Lei et al., 2022). The surface roughness of the salivary film increased significantly with the EGCG concentration, while EGC did not affect the surface morphology of the salivary film. High concentrations of EGCG can induce the aggregation of salivary proteins into large aggregates, while EGC cannot cause the aggregation of salivary proteins. A previous study using catechin and tannic acid to treat the salivary film also showed similar results. The thickness and surface roughness of the salivary film increased, causing lubrication loss and astringency (Lei et al., 2022).

The lubricity and wrinkling of the salivary film are influenced by its weak viscoelasticity, which results in a rapid increase in frictional force during tribological testing (Wang et al., 2021). Therefore, changes in astringency can be reflected by measuring the viscoelasticity of salivary film through rheological analysis. In this study, the changes in the G′ and G″ values of saliva after the addition of EGCG or EGC were dynamically observed using the SAOS time-scanning mode. As shown in Fig. 4b, the G′ and G″ values of saliva increased with lower concentrations of EGCG (0.25 and 0.5 mg/ml) and increased with higher concentrations of EGCG (1.0 and 2.0 mg/mL). Low concentrations of EGCG can moderately promote the aggregation of salivary proteins and enhance the elastic gel network structure of the salivary film. As the concentration of EGCG increased, excessive aggregation of salivary proteins disrupted the elastic gel network structure (Zhou et al., 2024). However, the G′ and G″ values of saliva did not change significantly with the addition of EGC within the experimental concentration range, indicating that EGC did not change the elastic gel network structure of the salivary film. Rossetti et al. (2008) used interfacial rheology to measure the effect of epicatechin (a non-gallate-type catechin) on the saliva film and also observed no significant effect, which was consistent with the experimental results.

In conclusion, the interaction between EGCG and salivary proteins is strong and can promote the aggregation and precipitation of salivary proteins, increasing the surface roughness of the salivary film, reducing its viscoelasticity, and producing astringency. Compared with EGCG, EGC lacks a galloyl group in its molecular structure, resulting in a weaker interaction with salivary proteins and an inability to promote aggregation and the precipitation of salivary proteins. Therefore, the astringency of EGC cannot be perceived through the tactile stimulus pathway.

### 3.4 Differences in the binding of EGCG and EGC to astringency receptors

Different mechanisms are responsible for the perception of different astringent compounds. A stronger astringency is associated with the aggregation of proteins and changes in the lubricating properties of saliva, while a weaker astringency can be perceived based on receptor stimulation (Bajec and Pickering, 2008; Rudge et al., 2021; Schöbel et al., 2014). The aforementioned studies demonstrated that EGC cannot produce astringency by promoting the aggregation of salivary proteins and loss of salivary lubricating properties. Thus, the weaker astringency of EGC may be perceived by the stimulation of astringency receptors. For example, the 67LR is a potential oral cell receptor that can sense polyphenol astringency (Schwarz and Hofmann, 2008). The 67LR is a highly conserved multifunctional protein that functions as a receptor for various dietary polyphenolic compounds, such as EGCG and quercetin (Gopalakrishna et al., 2024). Moreover, the hTAS2R39 human taste receptor has been confirmed to respond to the astringency of catechins (Narukawa et al., 2011). It has been reported that both EGCG and EGC can active the hTAS2R39 receptor (Deng et al., 2022).

**Fig. 5:**
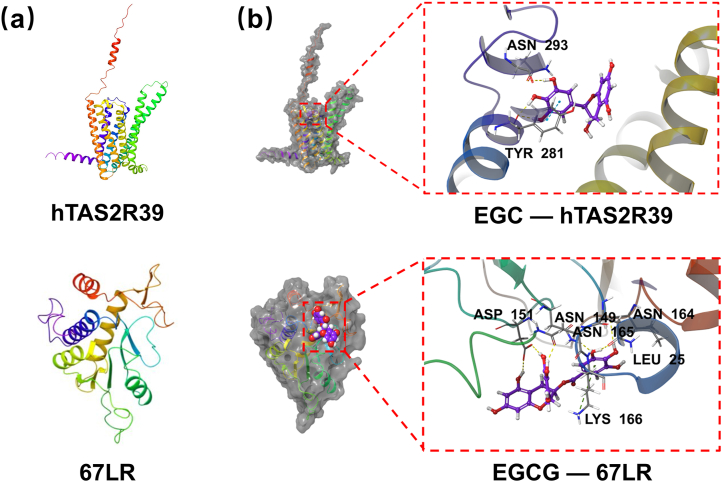
(a) The crystal structures of hTAS2R39 and 67LR. (b) The 3D images of EGCG and EGC docking with the 67LR and the hTAS2R39.

Using the 67LR and the hTAS2R39 human taste receptor as representative astringency receptors, molecular docking was employed to verify the binding of EGCG and EGC. The crystal structure of the hTAS2R39 human taste receptor protein (Fig. 5a) was generated by AlphaFold3, the pTM value of the structure predicted by AlphaFold3 is 0.78, and a value above 0.5 indicates that the overall prediction is similar to the true structure (Wee and Wei, 2024). The molecular docking results should refer to XP GScore and MM-GBSA dG Bind. When the XP GScore was less than −6, the ligand–protein binding was considered stable (Dong and Li, 2024; Gaur et al., 2025; Ivanova and Karelson, 2022). When the value of MM-GBSA dG Bind was less than −30 kcal/mol, the binding free energy was sufficiently low, further confirming the stability of the ligand–protein interactions (Wang et al., 2023). As shown in Table 1, the XP GScore and MM-GBSA dG Bind of EGC docking with the hTAS2R39 human taste receptor protein were −7.426 and −30.56, respectively, indicating the stable binding of EGC to the hTAS2R39 human taste receptor. Additionally, the interaction between EGCG and the 67LR protein had a sufficiently low docking score, with an XP GScore of −6.397 and an MM-GBSA dG Bind of −37.91. Both EGC and EGCG can induce astringency by stimulating receptors, which was consistent with our hypothesis.

**Table 1:** Thermodynamic parameters determined using ITC.

catechin	target	XP GScore	MM-GBSA dG Bind (kcal/mol)
EGC	hTAS2R39	−7.426	−30.56
EGCG		−6.137	−25.90
EGC	67LR	−4.134	−21.82
EGCG		−6.397	−37.91

The 3D images of EGCG and EGC docking with the 67LR protein and the hTAS2R39 human taste receptor protein are shown in Fig. 5b. EGC was deeply embedded within the active pocket of the hTAS2R39 human taste receptor protein, inducing hydrophobic interactions with the TYR104, TYR110, ILE288, LEU282, and TYR281 residues and forming hydrogen bonds with the ASN293 residue. It also formed two hydrogen bonds and a π–π bond with the TYR281 residue. EGCG was bound to the surface of the active pocket of the 67LR protein, generating hydrophobic interactions with the LEU25 residue and forming hydrogen bonds with the ASN165, ASN164, ASN149, LEU25, and ASP151 residues. Additionally, a π-cation bond was formed with the LYS166 residue.

It is worth noting that in the docking study an implicit solvent model was used. The model treats the solvent as a continuous medium without the need to explicitly simulate each solvent molecule. This approach offers significant advantages in computational efficiency. When dealing with some general π-π and π-cation interactions, it can provide relatively rapid estimations, which have certain reference value for preliminary screening and qualitative analysis. However, the π-π and π-cation interactions are often overestimated in docking studies without solvent corrections. The contribution of these interactions to overall binding energetics is worth further investigation in the future. Additionally, molecular docking alone may not capture the dynamic nature of ligand-receptor interactions. Performing molecular dynamics (MD) simulations could provide more robust insights into the binding stability and conformational flexibility (Sharma et al., 2023). In the future, MD simulations can be used to obtain more information about catechins stimulating astringency receptors.

## 4 Conclusion

**Fig. 6:**
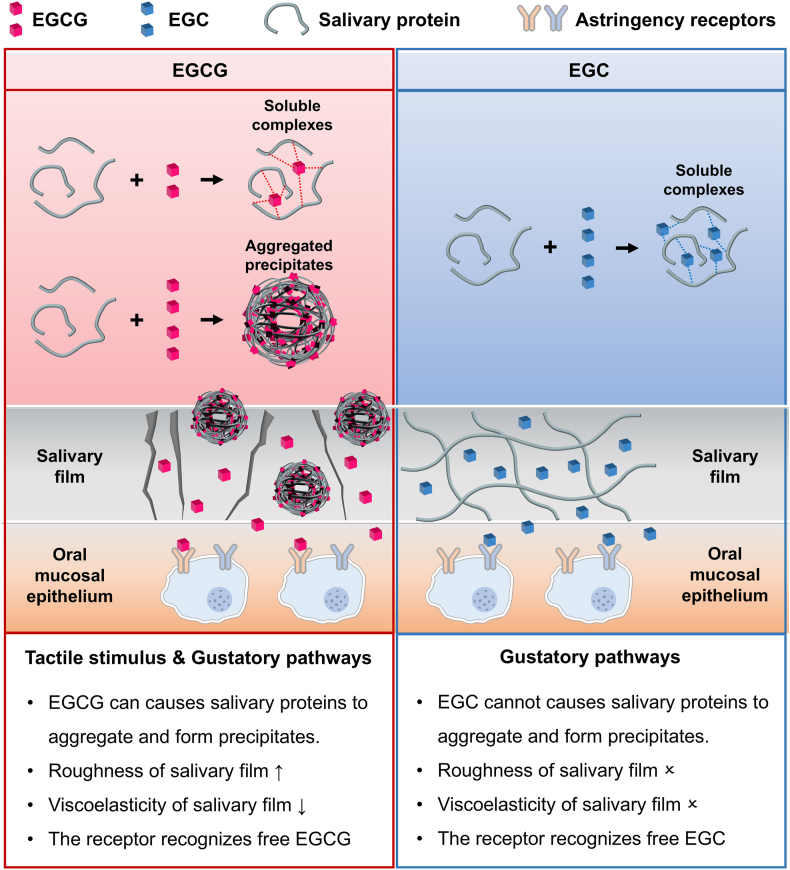
Schematic illustration of the mechanism of astringency perception by EGCG and EGC.

The C3 oxygen atom of EGCG is linked to a galloyl group, which is the key structural feature that distinguishes EGCG from EGC. This additional galloyl group accounts for the differences in their chemical reactivity, which consequently affects their binding capabilities with biological targets. The galloyl group of EGCG can form additional hydrogen bonds and hydrophobic interactions with salivary proteins. This creates a relatively stable hydrophobic core, which causes the surrounding protein molecules to continuously aggregate and form precipitates. Therefore, EGCG can increase the roughness of the salivary film and decrease its viscoelasticity, enabling the perception of astringency through the tactile stimulus pathway. The strong binding and high stability of EGCG to the 67LR protein suggest that the astringency of EGCG can also be perceived through the gustatory pathway. In contrast, the interactions between EGC and salivary proteins are weak and cannot alter the roughness or viscoelasticity of salivary film. Moreover, EGC can stably bind to the hTAS2R39 human taste receptor protein, so its astringency can only be perceived through the gustatory pathway (Fig. 6). This study not only elucidates the differences in the mechanisms of perceived astringency between gallate-type and non-gallate-type catechins but also provides a deeper understanding of the molecular basis of astringent compounds, thereby laying a theoretical foundation for the regulation of astringency.

## CRediT authorship contribution statement

**Zhenyu Zhou:** Conceptualization, Methodology, Investigation, Software, Data curation, Visualization, Writing - original draft. **Qian Huang:** Conceptualization, Methodology, Writing - review & editing. **Wangyang Shen:** Conceptualization, Methodology. **Weiping Jin:** Conceptualization, Methodology. **Guoyan Yang:** Conceptualization, Methodology. **Wenjing Huang:** Conceptualization, Methodology. **Cheng Guo:** Conceptualization, Methodology, Resources, Supervision, Validation, Project administration, Funding acquisition, Writing - review & editing.

## Declaration of competing interest

The authors declare that they have no known competing financial interests or personal relationships that could have appeared to influence the work reported in this paper.

## Data Availability

Data will be made available on request.
