# Supplementary material for: Effects of galloyl group on the astringency perception of epigallocatechin gallate and epigallocatechin
Source: Curr Res Food Sci. 2025 Jun 18;11:101120. doi: 10.1016/j.crfs.2025.101120 (PMC12226114; doi:10.1016/j.crfs.2025.101120)
Supplement: Multimedia component 1 [file mmc1.docx]

| catechin | combine | distance | category | type |
| --- | --- | --- | --- | --- |
| EGCG | EGCG:O – ARG195:HH22 | 2.58 | Hydrogen Bond | Conventional Hydrogen Bond |
|  | ILE235:HN – EGCG:O | 1.96 | Hydrogen Bond | Conventional Hydrogen Bond |
|  | HIS299:HE2 – EGCG:O | 2.64 | Hydrogen Bond | Conventional Hydrogen Bond |
|  | HIS299:HE2 – EGCG:O | 1.97 | Hydrogen Bond | Conventional Hydrogen Bond |
|  | EGCG:H – HIS305:O | 2.97 | Hydrogen Bond | Conventional Hydrogen Bond |
|  | EGCG:H – ASP300:O | 2.17 | Hydrogen Bond | Conventional Hydrogen Bond |
|  | EGCG:H – ASP197:OD1 | 1.84 | Hydrogen Bond | Conventional Hydrogen Bond |
|  | EGCG:H – ASP300:OD1 | 2.07 | Hydrogen Bond | Conventional Hydrogen Bond |
|  | EGCG:H – ASP300:OD1 | 1.99 | Hydrogen Bond | Conventional Hydrogen Bond |
|  | EGCG:H – GLU233:O | 2.28 | Hydrogen Bond | Conventional Hydrogen Bond |
|  | HIS201:NE2 – EGCG | 3.68 | Electrostatic | Pi–Cation |
|  | ASP300:OD2 – EGCG | 4.58 | Electrostatic | Pi–Anion |
|  | ASP300:OD2 – EGCG | 3.09 | Electrostatic | Pi–Anion |
|  | LEU162 – EGCG | 5.28 | Hydrophobic | Pi–Alkyl |
|  | HIS201 – EGCG | 5.38 | Hydrophobic | Pi–Alkyl |
|  | EGCG – LYS200 | 4.89 | Hydrophobic | Pi–Alkyl |
|  | EGCG – ILE235 | 4.7 | Hydrophobic | Pi–Alkyl |
| EGC | A:LYS200:NZ - EGC:O | 3.05 | Hydrogen Bond | Conventional Hydrogen Bond |
|  | EGC:H - A:ASP300:OD1 | 1.79 | Hydrogen Bond | Conventional Hydrogen Bond |
|  | EGC:H - A:HIS305:O | 2.69 | Hydrogen Bond | Conventional Hydrogen Bond |
|  | EGC:H - A:GLU233:O | 2.43 | Hydrogen Bond | Conventional Hydrogen Bond |
|  | A:VAL234:CA - EGC:O | 3.73 | Hydrogen Bond | Carbon Hydrogen Bond |
|  | A:ASP300:OD2 - EGC | 4.32 | Electrostatic | Pi-Anion |
|  | A:HIS201 - EGC | 4.64 | Hydrophobic | Pi-Pi T-shaped |
|  | EGC - A:LYS200 | 4.78 | Hydrophobic | Pi-Alkyl |
|  | EGC - A:ILE235 | 4.59 | Hydrophobic | Pi-Alkyl |

**Table S1.** The analysis of interaction forces between EGCG/EGC and α-amylase.

| catechin | combine | distance | category | type |
| --- | --- | --- | --- | --- |
| EGCG | A:ASN100:HN – EGCG:O | 2.71 | Hydrogen Bond | Conventional Hydrogen Bond |
|  | A:LEU101:HN – EGCG:O | 2.09 | Hydrogen Bond | Conventional Hydrogen Bond |
|  | EGCG:H – A:LYS96:O | 1.91 | Hydrogen Bond | Conventional Hydrogen Bond |
|  | EGCG:H – A:SER97:O | 2.16 | Hydrogen Bond | Conventional Hydrogen Bond |
|  | EGCG:H – A:SER53:O | 1.86 | Hydrogen Bond | Conventional Hydrogen Bond |
|  | EGCG:H – A:LYS96:O | 1.87 | Hydrogen Bond | Conventional Hydrogen Bond |
|  | EGCG:H – A:ASP102:OD2 | 1.9 | Hydrogen Bond | Conventional Hydrogen Bond |
|  | A:HIS49:NE2 – EGCG | 3.63 | Electrostatic | Pi–Cation |
|  | A:HIS49 – EGCG | 4.39 | Hydrophobic | Pi–Pi T–shaped |
|  | A:TYR64 – EGCG | 5.28 | Hydrophobic | Pi–Pi T–shaped |
|  | A:LEU101 – EGCG | 5.43 | Hydrophobic | Pi–Alkyl |
|  | A:HIS49 – EGCG | 5.09 | Hydrophobic | Pi–Alkyl |
|  | EGCG – A:PRO99 | 5.04 | Hydrophobic | Pi–Alkyl |
|  | EGCG – A:LEU101 | 5.29 | Hydrophobic | Pi–Alkyl |
|  | EGCG – A:LEU101 | 5.43 | Hydrophobic | Pi–Alkyl |
| EGC | A:ASP102:N - EGC:O | 3.12 | Hydrogen Bond | Conventional Hydrogen Bond |
|  | EGC:H - A:ASN100:OD1 | 1.88 | Hydrogen Bond | Conventional Hydrogen Bond |
|  | EGC:H - A:ASP102:OD2 | 1.88 | Hydrogen Bond | Conventional Hydrogen Bond |
|  | EGC:H - A:GLN98:O | 2.01 | Hydrogen Bond | Conventional Hydrogen Bond |
|  | EGC:H - A:LYS96:O | 1.84 | Hydrogen Bond | Conventional Hydrogen Bond |
|  | EGC:H - A:SER53:OG | 2.1 | Hydrogen Bond | Conventional Hydrogen Bond |
|  | A:LEU101:CD1 - EGC | 3.72 | Hydrophobic | Pi-Sigma |
|  | A:TYR64 - EGC | 5.74 | Hydrophobic | Pi-Pi T-shaped |

**Table S2.** The analysis of interaction forces between EGCG/EGC and cystatin S.

**Table S3.** The analysis of interaction forces between EGCG/EGC and lipocalin 1.

| catechin | combine | distance | category | type |
| --- | --- | --- | --- | --- |
| EGCG | EGCG:H – VAL113:O | 2.63 | Hydrogen Bond | Conventional Hydrogen Bond |
|  | EGCG:H – PHE99:O | 2.26 | Hydrogen Bond | Conventional Hydrogen Bond |
|  | EGCG:H – SER101:OG | 1.82 | Hydrogen Bond | Conventional Hydrogen Bond |
|  | EGCG:H – GLU34:OE1 | 2.04 | Hydrogen Bond | Conventional Hydrogen Bond |
|  | EGCG:H – VAL36:O | 1.97 | Hydrogen Bond | Conventional Hydrogen Bond |
|  | HIS84:CE1 – EGCG:O | 2.9 | Hydrogen Bond | Carbon Hydrogen Bond |
|  | ALA86:CB – EGCG | 3.96 | Hydrophobic | Pi–Sigma |
|  | LYS114:CE – EGCG | 3.33 | Hydrophobic | Pi–Sigma |
|  | MET39:SD – EGCG | 5 | Other | Pi–Sulfur |
|  | EGCG – PHE99 | 4.26 | Hydrophobic | Pi–Pi Stacked |
|  | GLU34:C,O;SER35:N – EGCG | 4.88 | Hydrophobic | Amide–Pi Stacked |
|  | EGCG – LYS114 | 4.13 | Hydrophobic | Pi–Alkyl |
|  | HIS84 – EGCG | 5.5 | Hydrophobic | Pi–Alkyl |
| EGC | A:THR16:N - EGC:O | 3.1 | Hydrogen Bond | Conventional Hydrogen Bond |
|  | A:THR16:OG1 - EGC:O | 2.9 | Hydrogen Bond | Conventional Hydrogen Bond |
|  | EGC:H - A:TYR18:OH | 1.89 | Hydrogen Bond | Conventional Hydrogen Bond |
|  | EGC:H - A:ARG118:O | 2.88 | Hydrogen Bond | Conventional Hydrogen Bond |
|  | EGC:H - A:ARG118:O | 1.88 | Hydrogen Bond | Conventional Hydrogen Bond |
|  | EGC:H - A:THR42:OG1 | 2.43 | Hydrogen Bond | Conventional Hydrogen Bond |
|  | A:GLY15:CA - EGC:O | 3.41 | Hydrogen Bond | Carbon Hydrogen Bond |
|  | A:THR16:CB - EGC | 3.86 | Hydrophobic | Pi-Sigma |
|  | A:SER14:C,O;GLY15:N - EGC | 4.31 | Hydrophobic | Amide-Pi Stacked |
